# BRAF inhibition causes resilience of melanoma cell lines by inducing the secretion of FGF1

**DOI:** 10.1038/s41389-018-0082-2

**Published:** 2018-09-20

**Authors:** Johannes Grimm, Anita Hufnagel, Marion Wobser, Andreas Borst, Sebastian Haferkamp, Roland Houben, Svenja Meierjohann

**Affiliations:** 1 Department of Physiological Chemistry, Biocenter, Würzburg Germany; 2 Department of Dermatology, Venereology and Allergology, University Hospital Würzburg, Würzburg, Germany; 3 Department of Dermatology, University Hospital Regensburg, Regensburg, Germany; 4 Comprehensive Cancer Center Mainfranken, University Hospital Würzburg, Würzburg, Germany

## Abstract

Approximately half of all melanoma patients harbour activating mutations in the serine/threonine kinase BRAF. This is the basis for one of the main treatment strategies for this tumor type, the targeted therapy with BRAF and MEK inhibitors. While the initial responsiveness to these drugs is high, resistance develops after several months, frequently at sites of the previously responding tumor. This indicates that tumor response is incomplete and that a certain tumor fraction survives even in drug-sensitive patients, e.g., in a therapy-induced senescence-like state. Here, we show in several melanoma cell lines that BRAF inhibition induces a secretome with stimulating effect on fibroblasts and naive melanoma cells. Several senescence-associated factors were found to be transcribed and secreted in response to BRAF or MEK inhibition, among them members of the fibroblast growth factor family. We identified the growth factor FGF1 as mediator of resilience towards BRAF inhibition, which limits the pro-apoptotic effects of the drug and activates fibroblasts to secrete HGF. FGF1 regulation was mediated by the PI3K pathway and by FRA1, a direct target gene of the MAPK pathway. When FGFR inhibitors were applied in parallel to BRAF inhibitors, resilience was broken, thus providing a rationale for combined therapeutical application.

## Introduction

The treatment of metastatic melanoma is currently based on two main pillars: targeted therapy addressing BRAF (v-Raf murine sarcoma viral oncogene homolog B)/MEK (Mitogen-activated protein kinase kinase) in BRAF-mutant melanoma patients, and immune therapy, applied irrespective of the driver mutation. For patients with BRAF-mutant tumors and a high tumor load, targeted therapy is frequently preferred, as therapy responsiveness occurs more quickly. Unfortunately, acquired as well as intrinsic resistance mechanisms limit the benefit of BRAF/MEK inhibitor therapy.

Mutational activation of the RAS (RAS viral oncogene homolog)/RAF (Rapidly Accelerated Fibrosarcoma kinase/MAPK (Mitogen activated protein kinase) pathways occurs in the majority of melanomas with acquired resistance. These mutations are the result of extended drug-induced selection processes. Most frequently, activating NRAS (Neuroblastoma RAS viral oncogene homolog), MEK1 and MEK2 mutations or BRAF amplifications are detected^1–4^. In contrast, intrinsic resistance is mostly caused by transcriptional rewiring of signaling pathways. Negative feedback regulators such as SPROUTY and SPRED family proteins are re-activated in response to MAPK inhibition, thereby increasing RAS activity and the responsiveness to growth factors^5,6^. Furthermore, the increased expression of receptor tyrosine kinases (RTK) like PDGFRB (Platelet derived growth factor receptor beta), EGFR (Epidermal growth factor receptor), MET (c-Met or hepatocyte growth factor receptor), and AXL (AXL receptor tyrosine kinase), which are induced due to the high phenotypic plasticity of melanomas and driven by diverse transcription factors, are correlated with reduced drug responsiveness^7–10^. In particular, high AXL expression, frequently in combination with low MITF (Microphthalmia transcription factor) levels, seems to predispose melanomas to resistance against BRAF/MEK inhibitors^11–13^.

But even in BRAF^V600E/K^ melanoma cells responding to BRAF inhibition, the anti-tumorigenic effect is limited, as apoptosis induction is incomplete. As a result, a fraction of melanoma cells survives, leading to disease relapse at the original metastatic sites^14^. Survival of cells under targeted therapy is likely favored by adaptive signaling crosstalk, which occurs under MAPK pathway inhibition and was shown to be beneficial for melanoma cell survival under stress conditions^5,15^. We and others have furthermore demonstrated that BRAF inhibition causes premature senescence in vitro and in vivo^16,17^. While senescence is generally considered anti-tumorigenic due to growth inhibition of the affected cell population, senescent cells have the potential to affect the surrounding tumor niche in a favorable manner. An enhanced secretory activity is one of the hallmarks of senescence. This senescence-associated secretory phenotype (SASP) leads to the secretion of cytokines and growth factors, which can—depending on the cellular context—positively or negatively affect tumor growth^18–20^.

In this study, we investigated the effect of BRAF/MEK inhibition in drug-responsive melanoma cells on the induction of SASP-like secreted factors. Our aim was the identification of targets, whose inhibition has the potential to improve anti-BRAF/MEK therapy.

## Results

### BRAF-inhibitor-conditioned medium favors cell growth

**Fig. 1:**
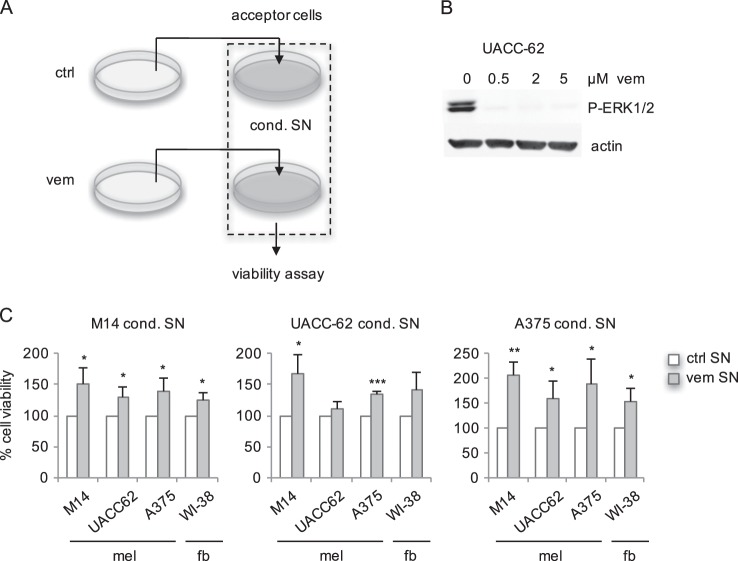
Treatment of melanoma cells with conditioned supernatant. **a** Conditioned supernatant (cond. SN) was generated from melanoma cells treated for 3 days with vemurafenib (vem, 0.5 µM) and from DMSO-treated control cells (ctrl). Donor cells were seeded to achieve an equal confluency at day 3. After excessive washing with PBS, donor cells were starved over night with medium containing 2% dialysed FCS. The following day, acceptor cells (which were starved for 3 days) were treated with filtered conditioned supernatant for 48 h followed by MTT measurement. **b** Western blot showing P-ERK1/2 (Thr202/Tyr204) of UACC-62 cells treated for 3 days with indicated concentrations of vemurafenib. Actin served as loading control. **c** MTT assays of the melanoma cell lines M14, UACC62, A375 and the fibroblast cell line WI-38 treated for 48 h with DMSO (“ctrl SN”) and vemurafenib-conditioned supernatant (“vem SN”). mel: melanoma; fb: fibroblast. Data are derived from three independent experiments. *p < 0.05; **p < 0.01; ***p < 0.001 (Student’s *t*-test, unpaired, comparison between vemurafenib-conditioned and control conditioned supernatant, which was set as 100%)

The secretion of factors under conditions of therapy stress harbours the potential to influence neighbouring cells in either positive or negative manners. In vivo, therapy-responsive melanoma cells are frequently accompanied by fibroblasts or by heterogenous populations of non-responsive melanoma cells, which coexist in the same tumor niche. To test the influence of BRAF inhibitor-induced factors on other cells, we developed a test system involving donor cells, which are treated with the BRAF inhibitor vemurafenib to generate vemurafenib-conditioned supernatant, and acceptor cells, which are treated with this conditioned supernatant (Fig. 1a). To avoid a negative effect of apoptotic donor cells on acceptor cells, donor cells were treated with 0.5 µM vemurafenib. At this concentration, apoptosis is reduced, while a strong senescence response^16^ and ERK1/2 inhibition (Fig. 1b) are observed. The three BRAF^V600E^ mutant cell lines UACC-62, M14, and A375 were used as donor cell lines. When conditioned medium from melanoma cells was added to vemurafenib-naive melanoma cells or the fibroblast-like cell line WI-38 as acceptor cell lines, vemurafenib-conditioned supernatant led to an enhanced viability of acceptor cells compared to control supernatant in almost all cases (Fig. 1c). As the absolute cell number also increased, as exemplarily shown for A375 conditioned supernatant (Supplementary Figure 1), we concluded that vemurafenib-conditioned supernatant has a growth-promoting effect on target cells.

### Transcription of pro-tumorigenic factors by BRAF inhibition

**Fig. 2:**
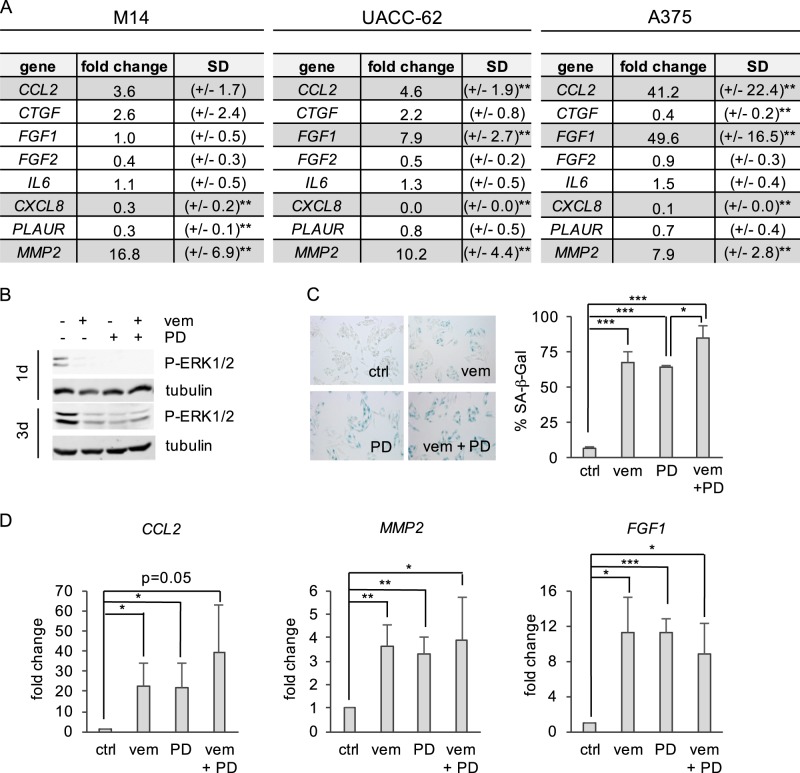
Expression of different secretome associated genes after BRAF-inhibitor or MEK inhibitor treatment. **a** Expression of different secretome associated genes after vemurafenib treatment. M14, UACC-62 and A375 were treated with vemurafenib or DMSO for 72 h. mRNA levels of the secretome associated genes were determined by real-time PCR. Gray color marks genes, which are regulated more than two-fold in at least two of the three cell lines and whose regulation reached significance. **p < 0.01 (Student’s test, unpaired, comparison with respective untretaed control). **b** Western blot showing P-ERK1/2 (Thr202/Tyr204) of A375 cells treated with vemurafenib (0.5 µM), PD184352 (0.5 µM) or both for one and three days. Tubulin served as loading control. **c** A375 cells were treated for 72 h with the MEK inhibitor PD184352 (0.5 µM) and/or vemurafenib (0.5 µM), after which SA-β-Gal staining was performed. Left: representative images; right: corresponding quantification (One-way ANOVA: *p* < 0.0001; post hoc test: t-test, unpaired, data derived from three independent experiments). **d** Corresponding expression of the secretome associated genes, as determined by real-time PCR (One-way ANOVA: *p* < 0.05, post hoc test: *t* test, unpaired, data derived from at least four independent experiments) *p < 0.05; **p < 0.01; ***p < 0.001. vem: vemurafenib. PD: PD184352

To test the influence of BRAF inhibition on SASP genes, we first generated a list of overlapping SASP genes, which are induced by various different senescence triggers^18,19,21,22^, and which includes cytokines, growth factors and proteases/protease receptors (Fig. 2a). The melanoma cell lines M14, UACC-62, and A375 were treated with vemurafenib for three days, and gene expression was monitored by real-time PCR. We focused on those genes, which were significantly regulated by vemurafenib by at least factor two, and which were regulated in at least two of the three cell lines. Four genes met these criteria. *CXCL8* (encoding interleukin 8) was strongly suppressed in all three cell lines. In contrast, CC-chemokine ligand 2 (*CCL2*) and matrix metalloprotease 2 (*MMP2*) were upregulated by BRAF inhibition in all cases, and fibroblast growth factor 1 (*FGF1*) was upregulated in two cell lines (Fig. 2a). Of note, CCL2, MMP2, and FGF1 have all been assigned pro-tumorigenic features.

In melanoma patients, dual BRAF/MEK inhibition has become standard of care treatment for BRAF^V600E/K^ positive patients due to the prolonged tumor response compared to BRAF inhibitor monotherapy^23^. We therefore tested senescence response and gene induction of *CCL2*, *MMP2*, and *FGF1* under conditions of single and combined BRAF and MEK inhibition in A375 cells. A solid downregulation of P-ERK/2 was observed in all cases (Fig. 2b). Importantly, senescence-associated β-galactosidase (β-Gal) staining and the induction of *CCL2*, *MMP2*, and *FGF1* were detected under all conditions (Fig. 2c, d). Similar observations were obtained for M14 cells (with the exception of *FGF1*, which is not induced in this cell line), and for UACC-62 cells, which showed a trend towards *CCL2* and *MMP2* upregulation as well as significant *FGF1* induction under conditions of BRAF and/or MEK inhibition (Supplementary Figure 2A–E). MMP2 was previously described to be induced in melanoma after MEK inhibition^24^, and we could confirm the secretion of the protease in the supernatant of vemurafenib-treated cells (Supplementary Figure 3A). Furthermore, the observed induction of *CCL2* and the reduction of *IL8* RNA expression were confirmed on protein level by ELISA in the supernatant of A375 cells (Supplementary Figure 3B).

### Deregulation of multiple fibroblast growth factors by BRAF inhibition

**Fig. 3:**
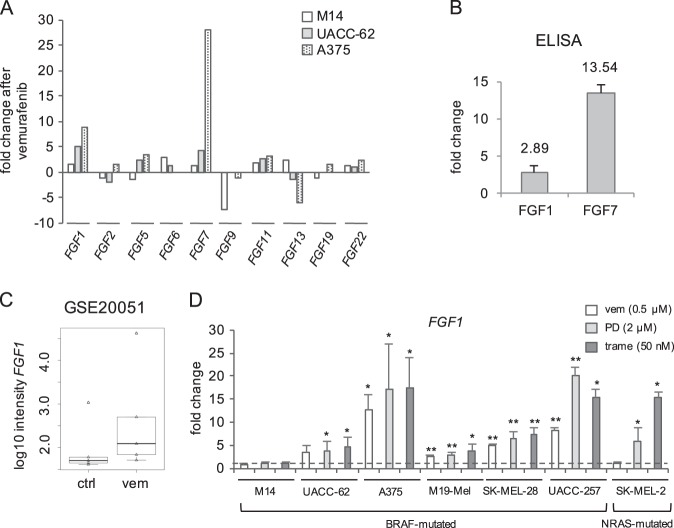
BRAF inhibition alters the FGF pathway. **a** Alterations of mRNA levels of indicated FGFR ligands, as measured by human growth factor PCR array, after treatment of M14, UACC-62, and A375 for 3 days with vemurafenib (0.5 μM). **b** Secretion of FGF1 and FGF7 in A375 cells treated with vemurafenib for 3 days (0.5 µM). Samples were measured by ELISA and are derived from two (FGF1) to three (FGF7) independent experiments. **c** Analysis of *FGF1* expression in the publicly available data set GSE20051 (*n* = 5) of melanoma cells exposed to 0.25 µM PLX4032 (vemurafenib) for 8 h. In the boxplot figure, the median value (in the box), the first and third quartile (upper and lower border of the box), and the minimum values are indicated. Maximum values are represented as outliers. **d** Real-time PCR of *FGF1* in indicated melanoma cell lines after 24 h of treatment with vemurafenib (0.5 µM) or the MEK inhibitors PD184352 (2 µM) or trametinib (50 nM). Data are derived from 2–5 independent experiments, each performed in triplicate, and are referred to control cells treated with the solvent DMSO. *p > 0.05; **p > 0.01

As FGFs are potent growth factors with multiple effects on different cell types of the melanoma microenvironment, we were interested to investigate if other FGFs are induced by BRAF inhibition in melanoma cells in addition to FGF1. To this end, we tested multiple cancer-relevant FGFs in the three melanoma cell lines using a growth factor PCR array. We could confirm the induction of *FGF1* in UACC-62 and A375 cells. Interestingly, a >2-fold upregulation was also detected for *FGF7* in these two cell lines (Fig. 3a), but not in M14 cells. In addition, we observed a weak induction of *FGF17* in all three cancer cell lines, though only at low levels, which could be detected by RT-PCR after extended rounds of amplification (Supplementary Figure 4A), but not by ELISA of either supernatant or cell lysate. Therefore, we excluded a relevant role of FGF17 for cellular resilience. In contrast, BRAF inhibitor-induced FGF1 and FGF7 secretion was clearly detected by ELISA, as exemplified in A375 cells (Fig. 3b). FGFs show different binding characteristics towards their high-affinity fibroblast growth factor receptors (FGFR) 1-4. FGF1 is able to bind all four receptors, while FGF7 only binds to FGFR2^25,26^. Semiquantitative analysis of cDNA from melanoma cells and the fibroblast cell line MainUro revealed that *FGFR1* is expressed in all analysed cell lines, while *FGFR2-4* are expressed variably (Supplementary Figure 4B). In a subsequent real-time PCR, we analysed the delta cT value between the respective FGFR and the housekeeping gene and confirmed these observations (Supplementary Figure 4C). This implies that FGF1 is most likely the relevant factor, which impacts melanoma cells as well as fibroblasts. BRAF-inhibitor-induced expression of *FGF1* was furthermore seen in an independent publicly available dataset using melanoma cell lines, which were treated with 0.25 µM vemurafenib for 8 h (Fig. 3c)^27^. However, the data did not reach statistical significance due to the high variations in expression levels between cell lines. To get better insight into a potential link between the RAF/MAPK pathway inhibition and *FGF1* expression in a larger set of melanoma cell lines, we treated M14, UACC-62, and A375 cell lines as well as four additional melanoma cell lines for 24 h with vemurafenib (0.5 µM) or the MEK inhibitors PD184352 (2 µM) and trametinib (50 nM), respectively (Fig. 3d). We chose this shorter treatment instead of our standard 3-day treatment, as the dataset from Joseph and colleagues indicated that the effect of BRAF inhibition on *FGF1* already occurs at early timepoints^27^. With the exception of M14 cells, *FGF1* was induced in all BRAF- but not NRAS-mutant cell lines in response to BRAF inhibition. Furthermore, MEK inhibition by either PD184352 or trametinib induced *FGF1* in six of seven cell lines, again with the exception of M14 cells. This was also observed for SK-MEL-2, the only NRAS-mutated cell line in this cohort, thus demonstrating that *FGF1* induction is a common response to MAPK pathway inhibition (Fig. 3d).

**Fig. 4:**
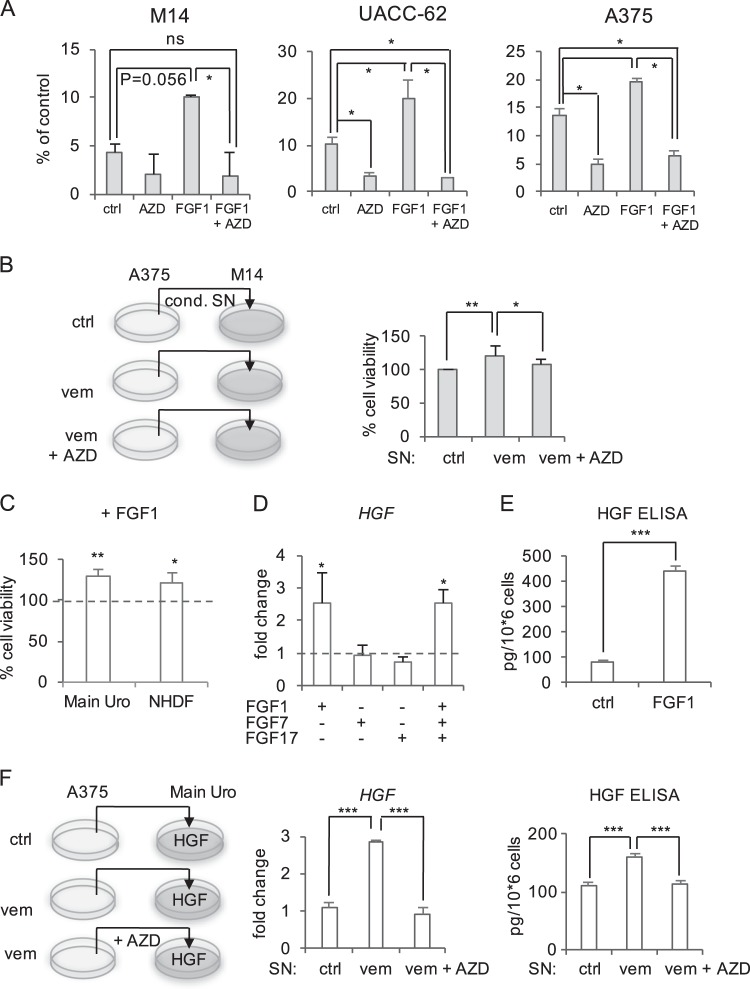
Paracrine effects of FGF1. **a** Sensitivity towards vemurafenib in presence of FGF1 and FGF inhibitor AZD4547. Cells were treated with vemurafenib (2 µM) in absence or presence of FGF1 (100 ng/ml) and AZD4547 (100 ng/ml) for 5 days. Medium was changed every 2 days. After 5 days, cells were counted. Data are derived from three independent experiments (One-way ANOVA: *p* < 0.05, post hoc test: *t* test, unpaired). **b** Viability of M14 melanoma cells, treated with control- or vemurafenib (0.5 µM)-conditioned supernatant of A375 cells for 2 days. Left image: scheme of the experimental setup. Right: corresponding quantification. Where indicated, FGFR inhibitor AZD (100 ng/µl) was added. (One-way ANOVA: *p* < 0.05, post hoc test: *t* test, unpaired). **c** MTT assay of MainUro fibroblasts and normal human dermal fibroblasts (NHDF) after treatment with recombinant FGF1 (100 ng/ml) for 5 days. Cells were starved in medium containing 2% FCS before FGF1 treatment. 1 × 10^3^ cells were seeded per 96 well and medium with the recombinant ligand was changed after 48 h. Significant differences are referred to the respective untreated control, which was set as 100%. **d** Real-time PCR of *HGF* in MainUro fibroblasts treated with FGF1, FGF7, FGF17 or a combination of these (100 ng/ml, 8 h). Data are normalized to the untreated controls and are derived from four independent experiments. **e** HGF secretion of MainUro cells after FGF1 treatment (100 ng/ml, 2 days), as measured by ELISA. Data are derived from three independent experiments. **f** Analysis of HGF secretion of MainUro fibroblasts in response to conditioned supernatant from melanoma cells. Left: Scheme of the experimental setup. Middle: *HGF* expression in MainUro cells treated for 2 days with conditioned control medium (ctrl) or vemurafenib-conditioned medium from A375 cells (vem) (see also Fig. 1). Where indicated, AZD4547 (100 nM) was added to MainUro cells (at the same time as vemurafenib-conditioned medium was applied). Right: Corresponding HGF secretion, as determined by ELISA. Data are derived from three independent experiments. Statistical analysis was done using One-way ANOVA (*p* < 0.0001; post hoc test: *t* test). *p < 0.05; **p < 0.01, ***p < 0.001

To test whether FGF1 affects the sensitivity of melanoma cells to BRAF inhibition, we treated the melanoma cell lines M14, UACC-62, and A375 for 5 days with 2 µM vemurafenib in absence or presence of AZD4547, FGF1 or a combination of both (Fig. 4a). In all cases, the cell number was significantly increased in presence of FGF1. The protective effect of FGF1 was reversed in presence of the FGFR inhibitor AZD4547. AZD4547 treatment alone reduced the cell number in UACC-62 and A375 cell lines. This effect was also seen in presence of 0.5 µM vemurafenib (Supplementary Figure 5A), but less pronounced. Even M14 cells, which show no induction of FGF1 in response to vemurafenib, were protected by exogenous FGF1 (Fig. 4a and Supplementary Figure 5A). However, AZD4547 alone only showed a weak effect on sensitivity towards vemurafenib, which did not reach significance under any vemurafenib concentration in M14 cells (compare “ctrl” and “AZD” in Fig. 4a and Supplementary Figure 5A). In absence of BRAF inhibition, cell viability was not affected by FGF1 (Supplementary Figure 5B). Moreover, the growth-promoting effect of vemurafenib-conditioned medium on melanoma cells was prevented when AZD4547 was applied simultaneously (Fig. 4b).

FGF1 has been described as potent mitogenic growth factor for fibroblasts. Accordingly, FGF1 significantly enhanced cell viability in MainUro and NHDF fibroblasts (Fig. 4c). Activated fibroblasts can be triggered to secrete microenvironment-remodeling factors, including HGF (hepatocyte growth factor), which plays a crucial role in mediating BRAF inhibitor resistance^28–30^. We therefore checked whether any of the FGFs, which are increased in response to BRAF inhibition, have an influence on HGF expression in fibroblasts. Recombinant human FGF1, but not FGF7 and FGF17, significantly induced *HGF* expression in fibroblasts (Fig. 4d). The same degree of *HGF* upregulation was observed when a combination of all three FGFs was applied. Importantly, FGF1-dependent HGF secretion was also detected on protein level (Fig. 4e). When vemurafenib-conditioned supernatant from A375 cells was applied to fibroblasts, HGF was also induced on transcriptional as well as on protein level (Fig. 4f). This induction was prevented in presence of the FGFR inhibitor AZD4547. Together, FGF1 has beneficial effects on melanoma cells and fibroblasts, thereby limiting the efficacy of BRAF inhibition.

### Involvement of PI3K and FRA1 in the transcription of secreted factors

**Fig. 5:**
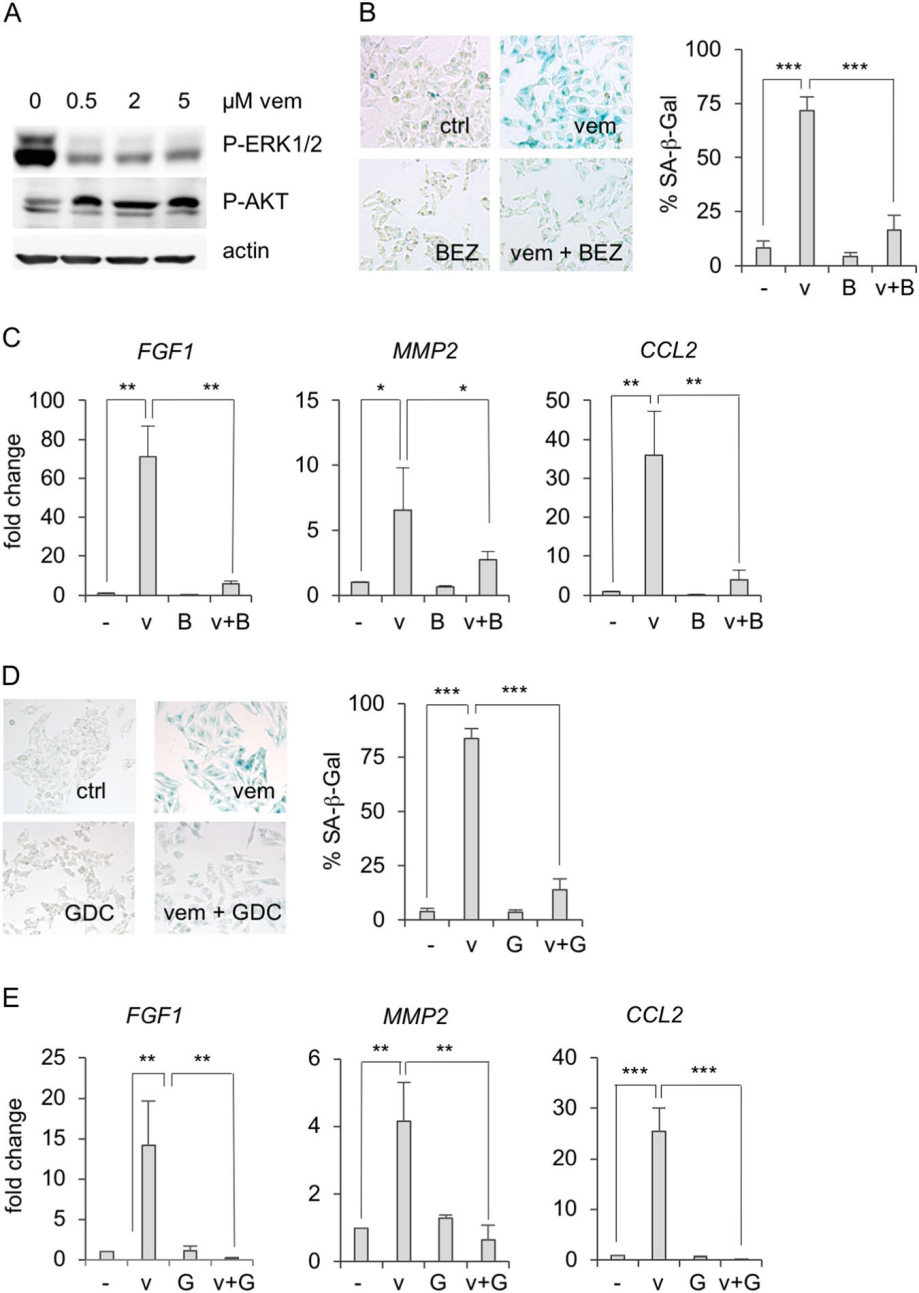
Involvement of PI3K pathway in vemurafenib-induced secretome. **a** Western blot of P-AKT (Ser473), P-ERK p42/44 (Thr202/Tyr204) and β-actin (loading control) in A375 cells, treated for 3 days with 0.5, 2 or 5 µM vemurafenib. **b** SA-β-Gal staining of A375 cells treated for 3 days with vemurafenib (0.5 µM) and/or the dual PI3K/mTOR inhibitor BEZ-235 (0.5 µM), as indicated. Left: representative images; right: corresponding quantification (One-way ANOVA: *p* < 0.0001; post hoc test: *t* test, unpaired, data derived from three independent experiments). **c** Corresponding real-time PCR of *FGF1*, *MMP2*, and *CCL2*. **d** SA-β-Gal staining of A375 cells treated for 3 days with vemurafenib (0.5 µM) and/or the PI3K inhibitor GDC-0941 (4 µM), as indicated. Left: representative images; right: corresponding quantification. **e** Corresponding real-time PCR of *FGF1*, *MMP2*, and *CCL2* (**d**, **e**: One-way ANOVA: *p* < 0.0001; post hoc test: *t* test, unpaired, data derived from three independent experiments). v: vemurafenib; B: BEZ-235; G: GDC-0941

BRAF or MEK inhibition in BRAF-mutant melanoma cells leads to immediate crosstalk mechanisms, including the RAS-mediated activation of the PI3K pathway^5,31,32^. This can be detected as an increase in phosphorylated AKT (Fig. 5a). To test if this compensatory pathway affects the expression of the secreted factors, we used the dual mTOR/PI3K inhibitor BEZ-235 (“BEZ”) in combination with vemurafenib. Interestingly, BEZ prevented the senescence-associated β-Gal staining by vemurafenib along with the induction of *FGF1*, *MMP2*, and *CCL2* (Fig. 5b, c). The PI3K inhibitor GDC-0941 led to the same effect (Fig. 5d, e), suggesting that the transcription of protective factors by BRAF inhibition is mediated by the PI3K pathway.

**Fig. 6:**
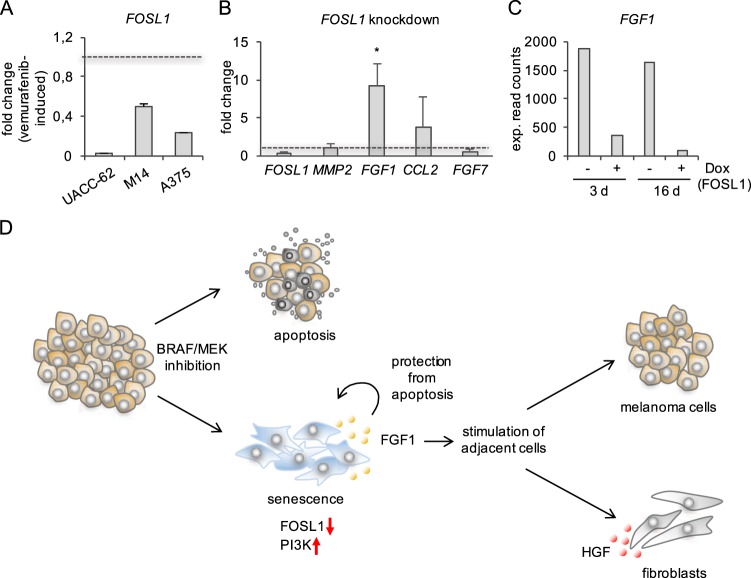
FRA1 as regulator of FGF1. **a** Real-time PCR of *FOSL1* in indicated melanoma cell lines after three days of vemurafenib treatment. Data are derived from two independent experiments and are referred to control cells treated with the solvent DMSO. **b** Real-time PCR of *FOSL1, MMP2*, a *FGF1*, *CCL2* and *FGF7* in UACC-62 cells after siRNA-mediated knockdown of *FOSL1* for 3 days, using smartpool siRNA. Data are derived from three different experiments. Significant differences are calculated between knockdown and the corresponding control siRNA experiment. **c** RNASeq results from melan-a FOSL1 cells, expressing doxycycline (Dox)-inducible *FOSL1* in response to Dox (1 µg/ml) for 3 and 16 days. Data are derived from^34^ (GEO accession number GSE85086). **d** Schematic overview of the FGF1-driven BRAF inhibitor resilience

In a previous study by Obenauf and colleagues, the influence of vemurafenib-responsive A375 cells on vemurafenib-resistant A375^R^ cells was investigated. In this context, the authors identified a set of genes, which was induced by vemurafenib treatment in A375 cells. They also observed *CCL2* induction, but no effect on *MMP2* or *FGF*s^33^. The authors reported that the AP-1 transcription factor component FRA1, which is transcribed and activated downstream of ERK1/2 and is suppressed by vemurafenib in our cell lines (Fig. 6a), is responsible for the detected gene induction^33^. We tested this hypothesis by siRNA-mediated knockdown in an independent melanoma cell line. Using UACC-62 cells, we analysed those genes, whose corresponding gene products were detectably secreted in our study, namely *MMP2*, *CCL2*, *FGF1*, and *FGF7*. Only FGF1 was significantly upregulated by siRNA-mediated knockdown of *FOSL1*, the gene encoding FRA1 (Fig. 6b). Recently, we described the effect of FRA1 expression on non-transformed melan-a melanocytes and performed transcriptome analysis of melan-a cells after 3 and 16 days of FRA1 expression^34^. Expression data from this analysis showed that *FGF1* is consistently repressed in response to FRA1, while *MMP2* and *CCL2* are not or inconsistently regulated by FRA1, and *FGF7* expression was too low for analysis (Fig. 6c and Supplementary Figure 6). These data demonstrate that *FGF1* is jointly induced by PI3K pathway and FRA1 repression, while PI3K, but not FRA1, contributes to the expression of *CCL2* and *MMP2*.

## Discussion

Inhibition of the BRAF/MEK pathway in melanomas constitutes an important treatment option for BRAF-mutant melanomas. Although BRAF-mutant drug-sensitive melanomas show increased apoptosis in response to BRAF or BRAF/MEK inhibition in vitro and in vivo, a sufficient number of cells frequently remains to allow the outgrowth of resistant melanomas at their original site^14,35^. One reason for tumor cell survival might be the fact that blocking BRAF/MEK causes premature senescence, a cellular state hard to attack by standard therapy^16,17^. Here we show that melanoma cells treated with the BRAF inhibitor vemurafenib secrete factors, which stimulate naive melanoma cells and fibroblasts, thereby limiting the anti-tumorigenic effect of BRAF inhibition. We identified FGF1 as novel mediator of BRAF inhibitor resilience (summarized in Fig. 6d).

The concept of a therapy-induced as well as a senescence-associated secretome was previously observed in melanoma. The chemotherapeutical agent cisplatin leads to a SASP, thereby exerting a positive effect on non-senescent melanoma cells^36^. Accordingly, premature senescence caused by the knockdown of the melanocyte lineage factor MITF stimulated a SASP, which had the capacity to convey melanoma cells with tumor initiating and metastatic features in a mouse model^20,22^. Interestingly, MITF knockdown also led to increased CCL-2 levels, which were involved in the tumor-promoting effect^20^.

Obenauf and collegues analysed the effect of vemurafenib-sensitive cell lines on vemurafenib-resistant cancer cells. They described that drug-sensitive A375 melanoma cells secrete various cytokines and stimulate growth and metastasis of drug-resistant A375 cancer cell clones^33^. Growth promotion of vemurafenib-resistant by vemurafenib-sensitive melanoma cells was furthermore confirmed with various cell lines. The coexistence of intrinsically drug-resistant as well as drug-sensitive melanoma cells at one site is a known phenomenon in clinical practice. It is due to the presence of heterogenous cell populations within one patient's tumor, e.g., cell clones harboring BRAF mutations and others comprising NRAS mutations. Our data now show that the secretome of vemurafenib-sensitive cells also has the potential to stimulate fibroblasts and naive melanoma cells. While this effect is most likely mediated by several factors, we identified FGF1 as one important contributing secretome component. FGF1 could limit the inhibitory effect of vemurafenib on melanoma cells, while the simultaneous inhibition of BRAF and FGF receptors increased the anti-tumorigenic effect. It was previously described that BRAF inhibition is able to induce the unfolded protein response (UPR) in melanoma cells^37^. Interestingly, another study recently demonstrated that the UPR can trigger the expression of FGF1 and FGF2 in melanoma cells^38^, thus raising the possibility that the BRAF inhibition causes UPR next to senescence and thereby leads to the secretion of protective FGF1. Potential differences in UPR between different cell lines could explain why some cells are potent FGF1 inducers after BRAF/MEK inhibitor treatment (e.g., A375 cells), why others are not (e.g., M14 cells).

Our study shows that BRAF inhibition triggers a response, which protects drug-responsive melanoma cells and thereby mediates resilience. In the tumor niche, this resilience can be further promoted by neighboring fibroblasts. We could show that FGF1 activates fibroblasts by triggering HGF secretion, which has previously been described as an important resistance-mediating factor in melanoma^28,29^. Although the majority of tested cell lines display FGF1 induction in response to BRAF or MEK inhibition, this was not observed in M14 cells. Still, the fact that vemurafenib-conditioned supernatant from M14 cells also stimulates melanoma cells and fibroblasts implies that other factors might play a protective role in this cell line. Indeed, preliminary analyses of other growth factor genes revealed that the ERBB4 ligand *NRG3* is induced by BRAF and MEK inhibitors in M14 cells (Supplementary Figure 7). The resulting effects will be the subject of future studies. However, this observation demonstrates that the upregulation of growth factors plays an even larger role than anticipated and might affect BRAF inhibitor resilience even when FGF1 is not regulated.

Various RTK such as IGF1R^39^, EGFR^10,40^, PDGFRα/β^41,42^, ERBB3^7^ or MET^29^ have been related to drug resistance in melanoma. These activated RTKs are an alternative means of MEK/ERK1/2 activation, thereby compensating for the loss of BRAF^V600E^ signaling in presence of BRAF/MEK inhibitors. In absence of other oncogenes, RTKs such as EGFR are able to convey potent pro-tumorigenic features to cells of melanocytic origin^43–46^. In contrast, BRAF^V600E/K^ positive melanoma cells tend to express undetectable or low levels of most RTKs, most likely because the strong endogenous MAPK activation by mutant BRAF selects against active RTK signaling to avoid senescence^10^. This is changed under drug pressure, where the expression of various RTKs is beneficial for survival.

The situation is different for FGFR1, which is expressed in the majority of melanomas, in contrast to FGFR2-4^47^(www.proteinatlas.org), thus suggesting that BRAF^V600E/K^ and FGFR1 expression do not exclude each other. The existence of FGFR1 expression prior to BRAF inhibition allows the melanoma cells to react immediately on the drug-induced ligands without the need for selection. This might pave the way for a persistant tumor cell population (“minimal residual disease”) which then may develop secondary mechanisms of resistance under continued BRAF inhibition. The therapeutical targeting of this core population is therefore an important aim for reaching a better overall response, which would be the complete elimination of all tumor cells in a best-case scenario, and avoiding the advent of resistance. Our data show that the PI3K pathway is involved in mediating the expression of FGF1, CCL2, and MMP2. A therapeutic combination of PI3K or FGFR inhibitors with BRAF/MEK inhibitors might therefore show enhanced anti-tumor effects. Combination therapies of BRAF/MEK inhibitors with PI3K-, CDK4/6-, MET-, and FGFR inhibitors are currently tested in clinical trials such as within the setting of the LOGIC-2 trial (https://clinicaltrials.gov/ct2/show/NCT02159066). However, the LOGIC-2 study design does not allow the up-front application of any of the additional inhibitors in a combinatorial approach, but only as add-on after progression from BRAF/MEK inhbitor therapy. At this time, the tumor cells are likely to have already undergone severe drug-triggered selection, which might impede the therapy success. A final evaluation will be possible after completion of the study.

PI3K inhibitors were repeatedly shown to work efficiently in pre-clinical models of melanoma, particularly in combination with BRAF and/or MEK inhibitors^48–50^. Unfortunately, most PI3K inhibitors are not well tolerated in tumor patients. Interestingly, FGFR inhibition was previously tested in a mouse melanoma model. The authors demonstrated that expression of dominant negative FGFR1 blocks melanoma growth in SCID mice and that this can be further enhanced by the multi-kinase inhibitor sorafenib^51^. In conclusion, we propose that the parallel inhibition of FGFR with BRAF/MEK inhibitors might be beneficial for melanoma patients due to the prevention of drug-induced and secretome-mediated resilience.

## Materials and methods

### Cell culture

Melanoma cells A375 and SK-MEL-28 were received from ATCC, while M14, UACC-62, M19-Mel, SK-MEL-2, and UACC-257 cells were obtained from the NCI/NIH (DCTD Tumor Repository, National Cancer Institute at Frederick, Frederick, MD). Cell line identity was confirmed by genotyping using PowerPlex 16 system (Promega, Mannheim, Germany). MainUro fibroblasts and WI-38 cells were received from the Department of Dermatology, Venereology and Allergology and the Department of Biochemistry and Molecular Biology, University of Würzburg, respectively. Normal human dermal fibroblasts (NHDF) were obtained from Promocell (Promocell, Heidelberg, Germany). Cells were cultivated in Dulbecco’s modified Eagle medium (DMEM) supplemented with 10% FCS and 1x penicillin/streptomycin (Sigma-Aldrich, Munich, Germany) at 37 °C and 5% CO_2_ for propagation. In all experiments containing inhibitors, medium and inhibitors were replaced after 48 h. BRAF inhibitor (vemurafenib, Axon Medchem, Groningen, Netherlands), MEK inhibitors (PD184352 or trametinib, both from Axon Medchem, Groningen, Netherlands), FGFR inhibitor (AZD 4547, Selleckchem, Munich, Germany), BEZ-235 (Axon Medchem, Groningen, Netherlands) and GDC-0941 (Selleckchem, Munich, Germany) were applied as indicated in the figure legends.

To determine the influence of FGF1 on cell lines, recombinant human protein was used. Cells were starved in 2% starving medium (DMEM with 2% dialysed FCS, Gibco/Thermo Fisher Scientific, Darmstadt, Germany) and treated for indicated timespans with 100 ng/ml recombinant FGF1 (Tebu-Bio, Offenbach, Germany).

### Generation of vemurafenib-conditioned supernatant

Conditioned supernatant was generated from indicated melanoma “donor” cells treated with 0.5 µM vemurafenib or with an equivalent amount of the solvent DMSO in the controls. After 3 days of treatment, cells were washed three times with PBS to remove residual vemurafenib from the medium, and cells were incubated over night with fresh “starving medium”, containing 2% dialysed FCS (Gibco/Thermo Fisher Scientific, Darmstadt, Germany) to generate conditioned medium. Notably, control cells and vemurafenib-treated cells were seeded at different cell densities, which were optimized for each cell line to reach a similar confluence after the three-day incubation period. The next day, the medium was filtered through 0.45 µm membrane filters and was subsequently applied to acceptor cells starved for three days in medium containing 2% dialysed FCS. In previous analyses, we found that a three-day starving period is optimal to sufficiently deprive cell lines of growth factors present in the serum in order to enable the responsiveness to auto- or paracrine growth factors^44^.

### ELISA

To measure the secreted factors, cell supernatant was concentrated approximately 20-fold with 10 kDa size exclusion centrifuge colums (Amicon Ultra-4, PLGC Ultracel-PL; Merck Millipore, Darmstadt, Germany). CCL2, IL-8, FGF7, FGF1, and HGF were measured with specific kits (R&D Systems, Woesbaden, Germany) according to the manufacturer’s recommendations. Cells with control medium (i.e., in absence of vemurafenib (melanoma cells) or FGF1 (MainUro cells)) were used as controls. Analysis was carried out using the Tecan microplate reader system.

### Cell lysis and western blot

Cells were lysed in lysis buffer (20 mM HEPES (pH 7.8); 500 mM NaCl, 5 mM MgCl_2_, 5 mM KCl; 0.1% deoxycholate, 0.5% Nonidet-P40; 10 µg/ml aprotinin; 10 µg/ml leupeptin; 200 µM Na_3_VO_4_; 1 mM phenylmethanesulphonyl-fluoride and 100 mM NaF). 30–50 µg of protein was separated by SDS-PAGE and analyzed by western blotting. Antibodies directed against β-actin, P-ERK p42/p44 (Thr202/Tyr204), P-AKT (Ser473), and MMP2 were received from Cell Signaling (Danvers, MA, USA) (Supplementary Table 1). The antibody targeting tubulin was obtained from Sigma (Taufkirchen, Germany).

### MTT assay

Cells were seeded in triplicates in 96-well plates and were allowed to attach overnight. The next day, treatment (conditioned medium or FGF1, as indicated in the respective figure legend) was started and cells were incubated for the indicated timespan, before 15 µl of a 5 mg/ml MTT solution (3-(4,5-dimethylthiazol-2-yl)-2,5-diphenyltetrazolium bromide) was added to each well and incubated for 2 h. Cells were then lysed with DMSO. The analysis of the resulting formazan accumulation was done according to the manufacturer’s recommendations (Sigma, Taufkirchen, Germany).

### Senescence-associated β-galactosidase assay

Cells were incubated under indicated conditions on 6 well-plates and were washed two times with PBS before staining. For fixation, cells were treated with 3.7% formaldehyde in PBS and were subsequently washed twice with PBS. One microliter of the staining solution (1 mg/ml X-Gal, 40 mM citric acid/sodium phosphate buffer (pH 6.0), 5 mM potassium ferricyanide, 5 mM potassium ferrocyanide, 150 mM NaCl, and 2 mM MgCl_2_) was added per 6-well and the plate was incubated at 37 °C with 5% CO_2_ for 16 h protected from light. After subsequent PBS washing steps, plates were kept at 4 °C until documentation by light microscopy.

### RNA extraction, cDNA synthesis, and real-time PCR

RNA isolation was performed using the RNEasy Kit (Qiagen, Hilden, Germany) according to the manufacturer’s protocol. RNA was reversely transcribed with a RevertAid First Strand cDNA Synthesis Kit (Thermo Fisher Scientific, Waltham, MA, USA). Fluorescence-based RT-qPCR was performed and analyzed with a Mastercycler ep Realplex (Eppendorf, Hamburg, Germany) using SYBR Green reagent (Life Technologies, Darmstadt, Germany). Gene expression was normalized to ACTB and calculated with the 2ΔΔcT method. Oligonucleotide sequences are indicated in Supplementary Table 2.

### siRNA transfection

Cells were treated with commercially available siRNA against human *FOSL1*, (siGENOME SMARTpool, Thermo Scientific (Dreieich, Germany) as well as control siRNA (ON-Target plus Non-Targeting pool, Thermo Scientific). X-treme gene transfection reagent (Roche, Mannheim, Germany) was applied for transfection according to the manufacturer’s recommendation. The following day, cells were reseeded for further experiments.

### Statistical analysis

Unless indicated otherwise, the graphs depict the mean values of at least three independent experiments, and standard deviations are indicated by error bars. One way ANOVA and Student’s *t*-test (two-tailed, unpaired) revealed statistical significance highlighted by asterisks (*p < 0.05; **p < 0.01, ***p < 0.001).

## Electronic supplementary material

Supplementary table 1

Supplementary table 2

Supplementary information
