# Supplementary material for: BRAF inhibition causes resilience of melanoma cell lines by inducing the secretion of FGF1
Source: Oncogenesis. 2018 Sep 20;7(9):71. doi: 10.1038/s41389-018-0082-2 (PMC6147791; doi:10.1038/s41389-018-0082-2)
Supplement: Supplementary file 1 — Supplementary table 1 [file 41389_2018_82_MOESM1_ESM.docx]

| **Oligonucleotide** | **ENSEMBL ID** | **Sequence** |
| --- | --- | --- |
| Actin-5` | ENSG00000075624 | 5`- GGCATCCTCACCCTGAAGTA-3` |
| Actin-3` |  | 5`- GGGGTGTTGAAGGTCTCAAA-3` |
| CCL2-5` | ENSG00000108691 | 5`-CAATGCCCCAGTCACCTGCTGT-3` |
| CCL2-3` |  | 5`-GGGTTTGCTTGTCCAGGTGGTCC-3` |
| CTGF-5` | ENSG00000118523 | 5`-GCGAGGAGTGGGTGTGTGACG-3` |
| CTGF-3` |  | 5`-AGCCTGCAGGAGGCGTTGTC-3` |
| CXCL8-5` | ENSG00000169429 | 5`-GAGTGGACCACACTGCGCCA-3` |
| CXCL8-3` |  | 5`-TGCTTGAAGTTTCACTGGCATCTTCA-3` |
| FGF1-5` | ENSG00000113578 | 5`-CAGCCCTGACCGAGAAGTTT-3` |
| FGF1-3` |  | 5`-GGTTCTCCTCCAGCCTTTCC-3` |
| FGF2-5` | ENSG00000138685 | 5`-TCCCGCCCGGCCACTTCAA-3` |
| FGF2-3` |  | 5`-GCCAGGTAACGGTTAGCACACACT-3` |
| FGF7-5` | ENSG00000140285 | 5`-CCCTGAGCGACACACAAGAA-3` |
| FGF7-3` |  | 5`-TTCCACCCCTTTGATTGCCA-3` |
| FGF17-5` | ENSG00000158815 | 5`-CCCAACCTCACTCTGTGCTT-3` |
| FGF17-3` |  | 5`-CAAACTTGTTGCCGTCCTCG-3` |
| FGFR1-5` | ENSG00000077782 | 5`-GACTCCGGCCTCTATGCTTG-3` |
| FGFR1-3` |  | 5`-CCAATATGGAGCTACGGGCA-3` |
| FGFR2-5` | ENSG00000066468 | 5`-CAAACGTATCCCCCTGCGG-3` |
| FGFR2-3` |  | 5`-TGCCCAGTGTCAGCTTATCTC-3` |
| FGFR3-5` | ENSG00000068078 | 5`-GGAGTTCCACTGCAAGGTGT-3` |
| FGFR3-3` |  | 5`-AAGGTGACGTTGTGCAAGGA-3` |
| FGFR4-5` | ENSG00000160867 | 5`-GGAGGAGCCAGGTGAGGA-3` |
| FGFR4-3` |  | 5`-CTGCTCTTGCTGCTCCAGG-3` |
| HGF-5` | ENSG00000019991 | 5`-ATCTCCTCCTGCTCCCCATC-3` |
| HGF-3` |  | 5`-GCCTTGCAAGTGAATGGAAGT-3` |
| IL-6-5` | ENSG00000136244 | 5`-GCCTTCCCTGCCCCAGTACCC-3` |
| IL-6-3` |  | 5`-TGCCTCTTTGCTGCTTTCACACATG-3` |
| MMP2-5` | ENSG00000087245 | 5`-ACCTAGCACATGCAATACCTGAACACC-3` |
| MMP2-3` |  | 5´-CACCAGTGCCTGGGGCGAAG-3` |
| NRG3-5` | ENSG00000185737 | 5`-ACCTCTGTTCATCGGCTTCA-3` |
| NRG3-3` |  | 5`-TGGGCTTGGAGAGGAAGAAG-3` |
| PLAUR-5` | ENSG00000011422 | 5´-GCAACGAGGGCCCAATCCTGG-3` |
| PLAUR-3` |  | 5`-GCGGTTGCACAGCCTCTTACCA-3` |
| RPS14-5` | ENSG00000164587 | 5`-CTCAGGTGGCTGAAGGAGAG-3` |
| RPS14-3` |  | 5`-GCAGCCAACATAGCAGCATA-3` |

In addition, expression of *FGF5, -6, -9, -11, -13, -19* and *-22* was determined with the Growth Factors RT2 ProfilerTM PCR Array (SA Biosciences, Qiagen, Hilden, Germany), as recommended by the manufacturer.
