# Supplementary material for: BRAF inhibition causes resilience of melanoma cell lines by inducing the secretion of FGF1
Source: Oncogenesis. 2018 Sep 20;7(9):71. doi: 10.1038/s41389-018-0082-2 (PMC6147791; doi:10.1038/s41389-018-0082-2)

Supplementary Material

Supplementary Figure 1: Induction of cell proliferation by vemurafenib-conditioned supernatant.
Conditioned supernatant (SN) was generated from A375 cells treated for 3 days with vemurafenib (see also Figure 1) and from DMSO-treated control cells (ctrl). Donor cells were seeded to achieve an equal confluency at day 3. After excessive washing with PBS, donor cells were starved over night with medium containing 2% dialysed FCS. The following day, starved acceptor cells were treated with filtered conditioned supernatant for 72 h followed by manual counting. Data are derived from two independent experiments performed in triplicates. *: $p<0.05$; **: $p<0.01$

Supplementary Figure 2: Vemurafenib-induced genes are dependent on the MAPK pathway
A: Western blot showing P-ERK1/2 (Thr202/ Tyr204) of M14 and UACC-62 cells treated with vemurafenib (0.5 µM), PD184352 (0.5 µM) or both for one and three days. Tubulin served as loading control. **B:** M14 cells were treated for 72 h with the MEK inhibitor PD184352 (0.5 µM) and/or vemurafenib (0.5 µM), after which SA-β-Gal staining was performed. Left: representative images; right: corresponding quantification (One-way ANOVA: $p<0.0001$; post hoc test: t test, unpaired, data derived from three independent experiments). **C:** Corresponding expression of *CCL2* and *MMP2* in M14 cells, as determined by real-time PCR (One-way ANOVA: $p<0.05$; post hoc test: t test, unpaired). **D:** As in B, but for UACC-62 cells. Left: representative images; right: corresponding quantification (One-way ANOVA: $p<0.0001$; post hoc test: t test, unpaired, data derived from three independent experiments). **E:** Corresponding expression of *CCL2*, *MMP2* and *FGF1* in UACC-62 cells, as determined by real-time PCR (One-way ANOVA: $p<0.05$; post hoc test: t test, unpaired). Real-time data are derived from at least 4 independent experiments. *: $p<0.05$; **: $p<0.01$; ***: $p<0.001$. vem: vemurafenib. PD: PD184352

Supplementary Figure 3: Expression of MMP2 in response to vemurafenib treatment
A: Protein blot with conditioned supernatant (MMP2) and whole cell lysate (P-ERK1/2; tubulin) of M14, UACC-62 and A375 cells treated for 3 days with indicated concentrations of vemurafenib. **B:** Secretion of CCL2 and IL8 in A375 cells treated with vemurafenib for 3 days (0.5 µM). Samples were measured by ELISA and are derived from two independent experiments.

Supplementary Figure 4: Expression of FGFR pathway components in different cell lines.
A: PCR of *FGF17* in M14, UACC-62 and A375 cells treated for 3 days with the MEK inhibitor PD184352 (0.5 µM) and vemurafenib (0.5 µM), as indicated (40 cycles). Samples were loaded on a 1% agarose gel. **B:** PCR (40 cycles) with cDNA of MainUro fibroblasts and the melanoma cell lines M14, UACC-62 and A375. PCR products were run on a 1% agarose gel. **C:** Expression of *FGFR1-4*, measured by real-time PCR. To allow a comparison of the expression between cell lines, quantification is shown by delta cT (*FGFR-RPS14*) values, where *"FGFR"* corresponds to the respective FGFR gene and *"RPS14"* is the housekeeping gene, which is always expressed at higher levels than the FGFRs and therefore exhibits a lower cT value. Please note that lower delta cT values indicate a higher expression. *ACTB* and *RPS14* served as controls, where indicated.

Supplementary Figure 5: FGF1 protects from vemurafenib
A: Sensitivity towards vemurafenib in presence of FGF1 and FGF inhibitor AZD4547. Cells were treated with vemurafenib (0.5 µM) in absence or presence of FGF1 (100 ng/ml) and AZD4547 (100 ng/ml) for 5 days. Medium was changed every 2 days. After 5 days, cells were counted. Data are derived from three independent experiments (statistical analysis by One-way ANOVA: $p<0.05$; post hoc test: t test, unpaired). *: $p<0.05$. **B:** Cell number of

indicated melanoma cell lines in presence of FGF1 (100 ng/ml) for 5 days. Data are normalized to the untreated condition. Cells were starved in medium containing 2% dialysed FCS before FGF1 treatment. Cells were quantified by manual counting. Data are derived from three independent experiments.

Supplementary Figure 6: Expression of *FGF7* and *CCL2* in response to FRA1 overexpression

RNASeq results from melan-a FOSL1 cells, expressing doxycycline-inducible FOSL1 in response to Dox (1 µg/ml) for 3 and 16 days. Data are derived from [1] (GEO accession number GSE85086).

Supplementary Figure 7: Induction of NRG3 in vemurafenib-treated M14 cells

Real-time PCR of *NRG3* in M14 melanoma cells treated with 0.5 or 2 µM vemurafenib for three days. *RPS14* served as reference gene. Data are derived from three independent experiments. Statistical differences were calculated between inhibitor-treated cells and the DMSO treated solvent control. *: p<0.05, **: p<0.01

References

1 Maurus K, Hufnagel A, Geiger F, Graf S, Berking C, Heinemann A *et al* (2017). The AP-1 transcription factor FOSL1 causes melanocyte reprogramming and transformation. *Oncogene* 36: 5110-5121.

Supplementary Figure 1

A

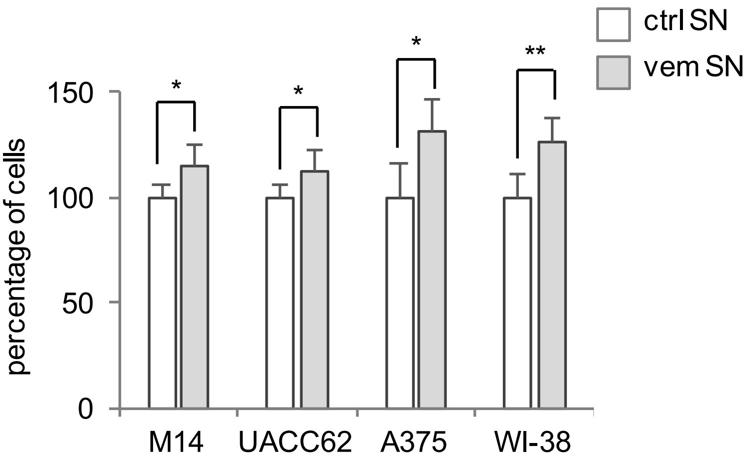

Supplementary Figure 2

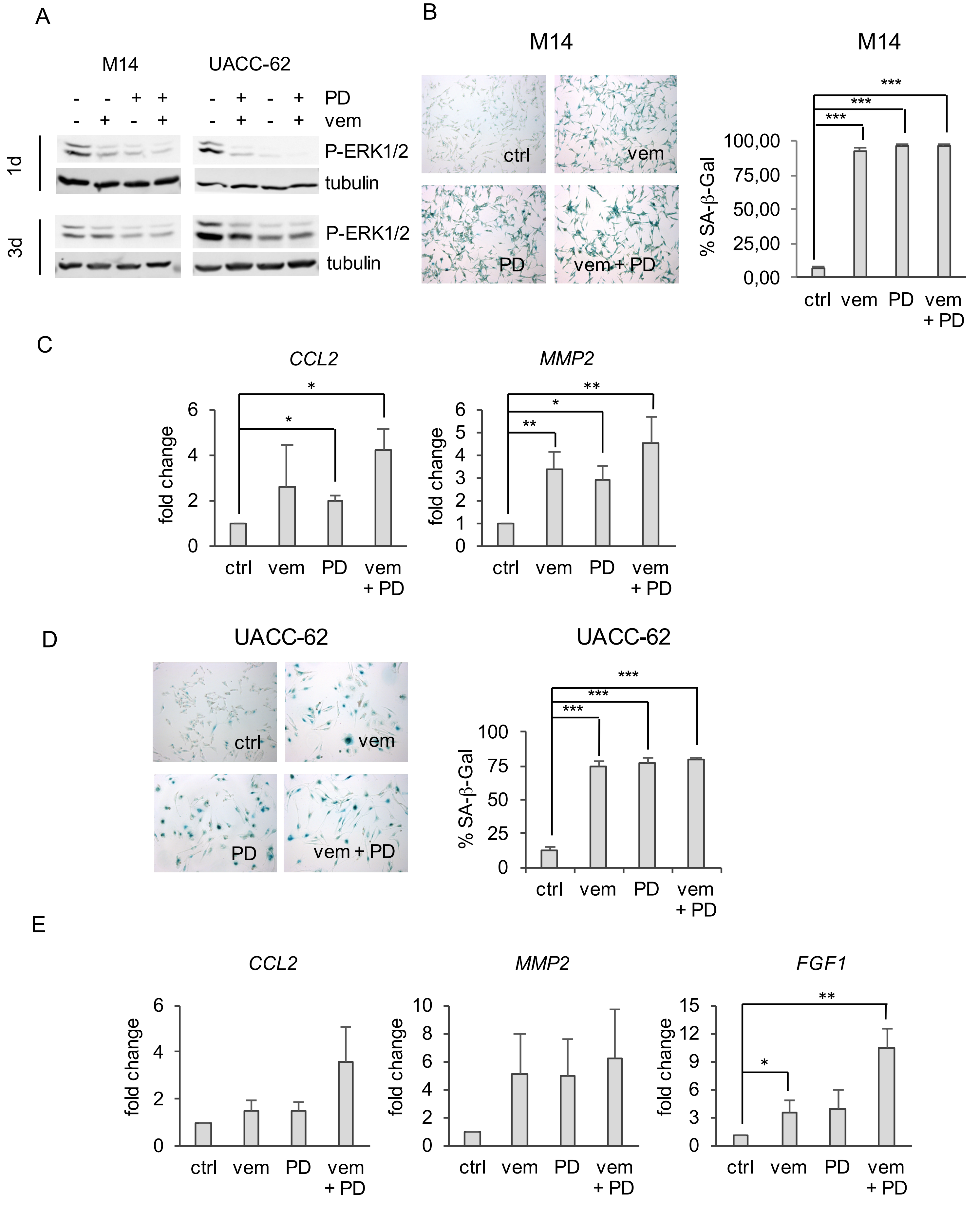

Supplementary Figure 3

A

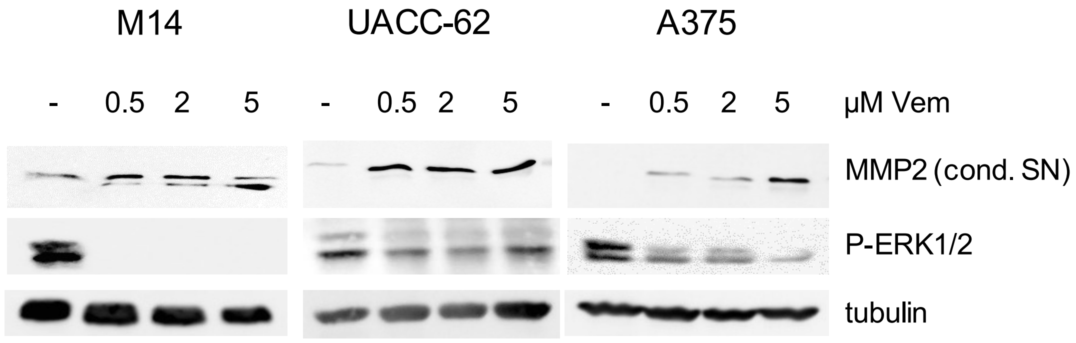

B

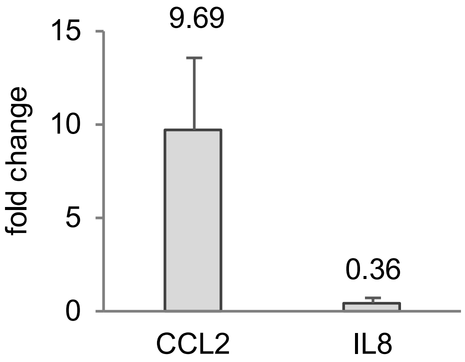

Supplementary Figure 4

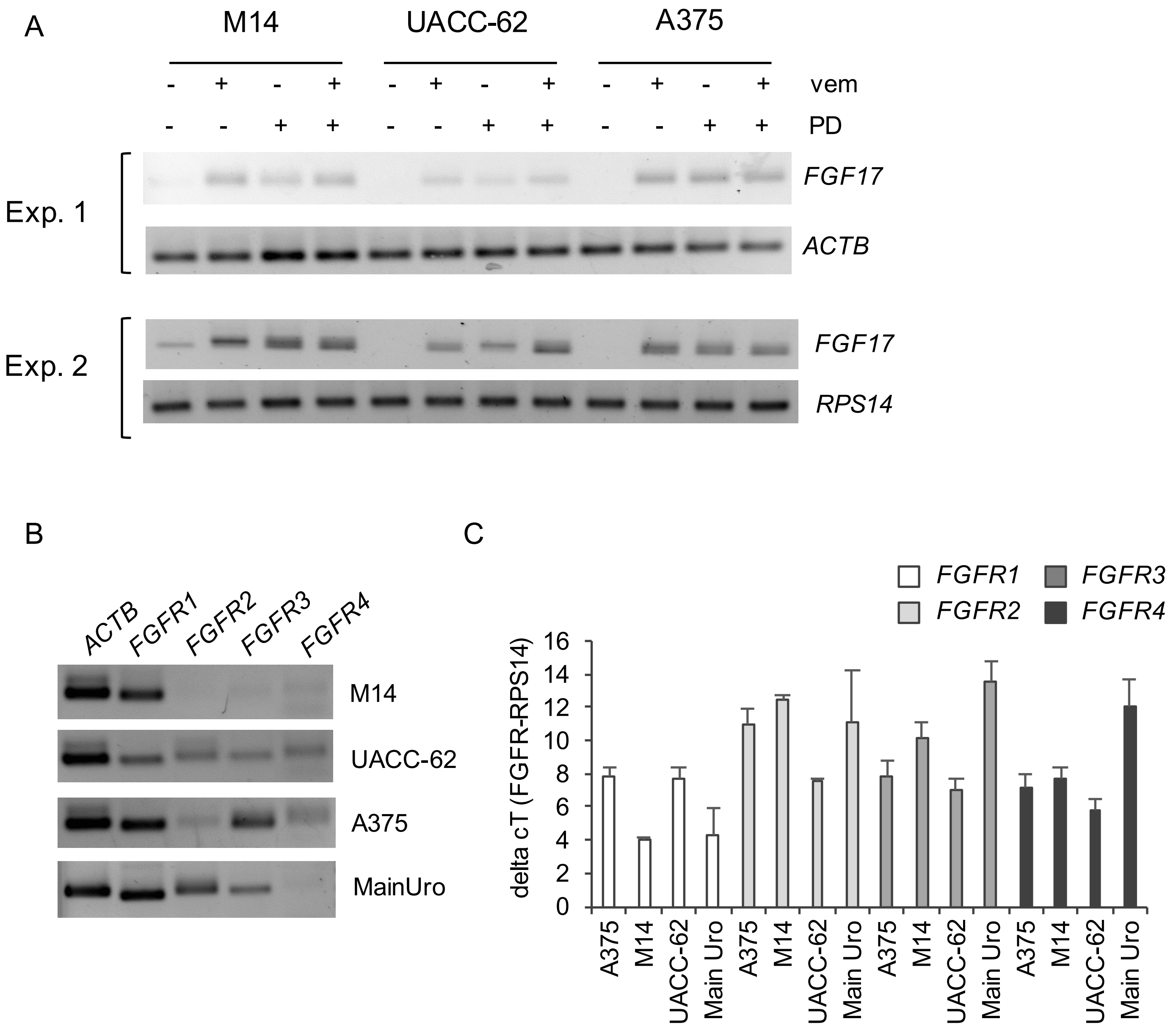

Supplementary Figure 5

A

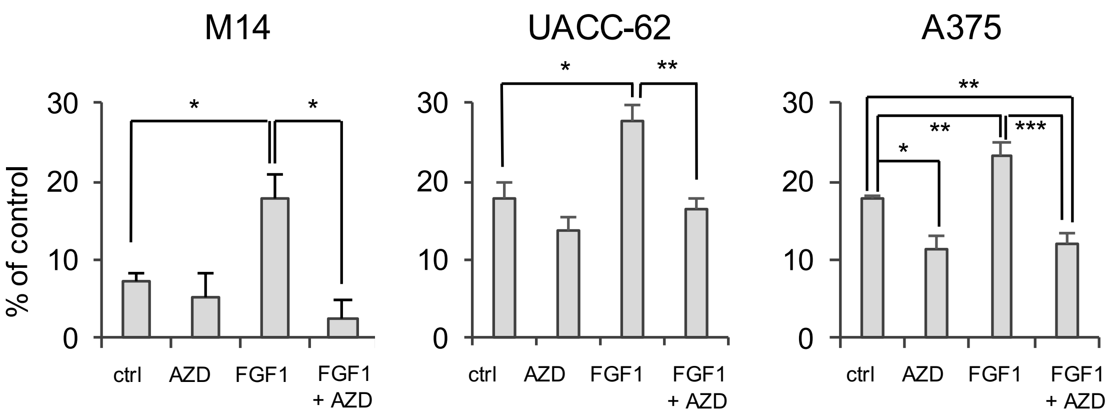

B

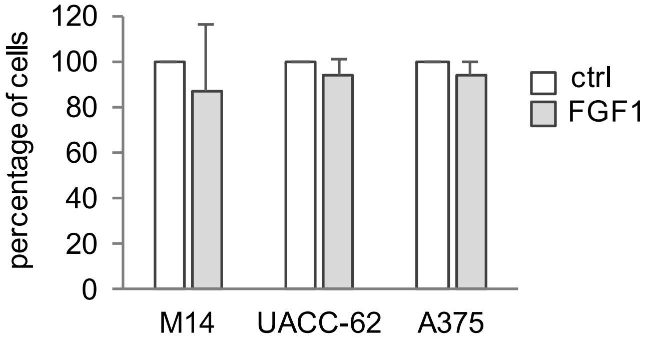

Supplementary Figure 6

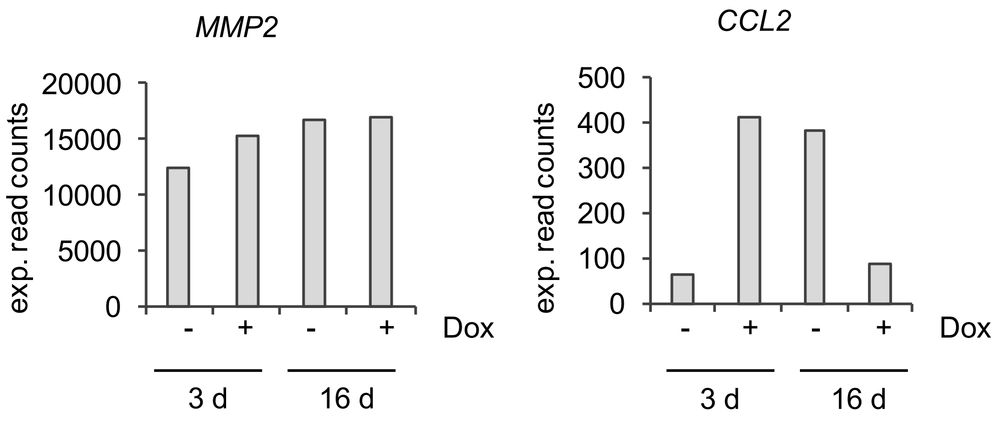

Supplementary Figure 7

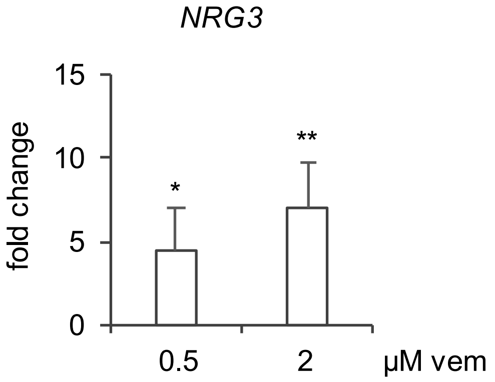

Supplement: Supplementary file 2 — Supplementary table 2 [file 41389_2018_82_MOESM2_ESM.pdf]
