# Supplementary material for: BRAF inhibition causes resilience of melanoma cell lines by inducing the secretion of FGF1
Source: Oncogenesis. 2018 Sep 20;7(9):71. doi: 10.1038/s41389-018-0082-2 (PMC6147791; doi:10.1038/s41389-018-0082-2)
Supplement: Supplementary file 3 — Supplementary information [file 41389_2018_82_MOESM3_ESM.docx]

| **Antibody** | **Company, product number, lot number** | **Dilution** |
| --- | --- | --- |
| β-Actin | Santa Cruz Biotechnology, sc47778, Lot F1011 | 1:5000 |
| P-ERK1/2 (Thr202/Tyr204) | Cell Signaling, #9101, Lot 28 | 1:5000 |
| P-AKT (Ser473) | Cell Signaling, #4060, Lot 13 | 1:2000 |
| MMP2 | Cell Signaling, #4022, Lot 2 | 1:1000 |
| Tubulin | Sigma, T6074, Lot 088K4829 | 1:10000 |
